# Research Publication Experience as a Requirement for Board Examination Acceptance to Promote Scholarly Activities of Pediatric Residents

**DOI:** 10.31662/jmaj.2021-0149

**Published:** 2021-12-28

**Authors:** Osamu Nomura, Nobuaki Michihata, Kazunari Kaneko, Tetsushi Yoshikawa, Akira Ishiguro, Japan Pediatric Society Steering Committee for Board Examinations

**Affiliations:** 1 Center for Postgraduate Education and Training, National Center for Child Health and Development, Tokyo, Japan; 2 Department of Emergency and Disaster Medicine, Hirosaki University, Aomori, Japan; 3 Department of Health Services Research, The University of Tokyo, Tokyo, Japan; 4 Department of Pediatrics, Kansai Medical University, Osaka, Japan; 5 Department of Pediatrics, Fujita Health University, Aichi, Japan

**Keywords:** Scholarly activity, publication, pediatric residency, board examination

## Abstract

**Introduction:**

Scholarship is an essential component of postgraduate education. This study’s objective was to investigate the effect of a new reform requiring research publication experience before taking the pediatric board examination to promote scholarly activities among pediatric residents in Japan.

**Methods:**

We conducted an experimental study from 2015 to 2018 to investigate the effectiveness of this reform for promoting scholarly activities among Japanese pediatric residents.

**Results:**

Of all 2524 participants, the number of examinees before and after the reform was 1580 and 944, respectively. The yearly number of the residents’ presentations and publications during their residency was 1.2 (*SD* 0.9) and 0.06 (*SD* 0.16), respectively, before the reform and 1.3 (*SD* 1.0) and 0.21 (*SD* 0.18), respectively, after the reform. Multiple regression showed the post-reform examinees (*β* = 0.37, *p* < 0.01) and the number of research presentations (*β* = 0.28, *p* < 0.01) were significantly associated with the number of research publications during the residency. While no contributive variables were found in the institution types, residents in the Kyushu and Okinawa area (i.e., southern island area in Japan) published fewer articles than those in the Tokyo area (*β* = −0.05, *p* = 0.03).

**Conclusions:**

The newly implemented policy requiring residents to publish research articles as a board examination prerequisite effectively promotes research activities among pediatric residents.

## Introduction

Promoting scholarly activities of residents can improve the quality of patient care by health care professionals and patient outcomes ^(1), (2)^. Accrediting organizations, such as the Accreditation Council for Graduate Medical Education in the United States and CanMeds in Canada, require that residency programs prepare a curriculum for supporting residents’ scholarly activities ^(3)^. Enhanced research activities also enable residents to enhance career progression as scientist physicians ^(4)^. Residency programs have adopted various approaches to satisfy the requirements and address the barriers, including protected research time, funding incentives, or mentorship programs ^(5), (6), (7)^. The combination of these strategies has been reported to be useful to promote scholarly activities among residents. However, no robust solutions have been established to overcome the potential barriers and increase scholarly activities of residents ^(8), (9)^.

To deal with this issue, the Japan Pediatric Society (JPS) launched a fundamental initiative that included publication experience in peer-reviewed journals as an examination eligibility criterion in 2017 ^(10)^. This study’s objective was to investigate the effect of the newly initiated policy requiring research publication before taking the pediatric board examination on promoting scholarly activities among pediatric residents in Japan.

## Materials and Methods

### Context

In Japan, all physicians must complete a 2-year rotation training under the regulation of the Ministry of Health, Labour and Welfare after graduating from medical school ^(11)^. JPS manages the board certification examinations, and trainees who choose pediatrics as a specialty enroll in a pediatric residency program approved by the JPS, which is supervised by a program director whose responsibility is to approve the trainee’s application for the board examination based on various prerequisites, including completion of a residency logbook and a case summary report ^(12)^.

### Design and participants

**Figure 1.**
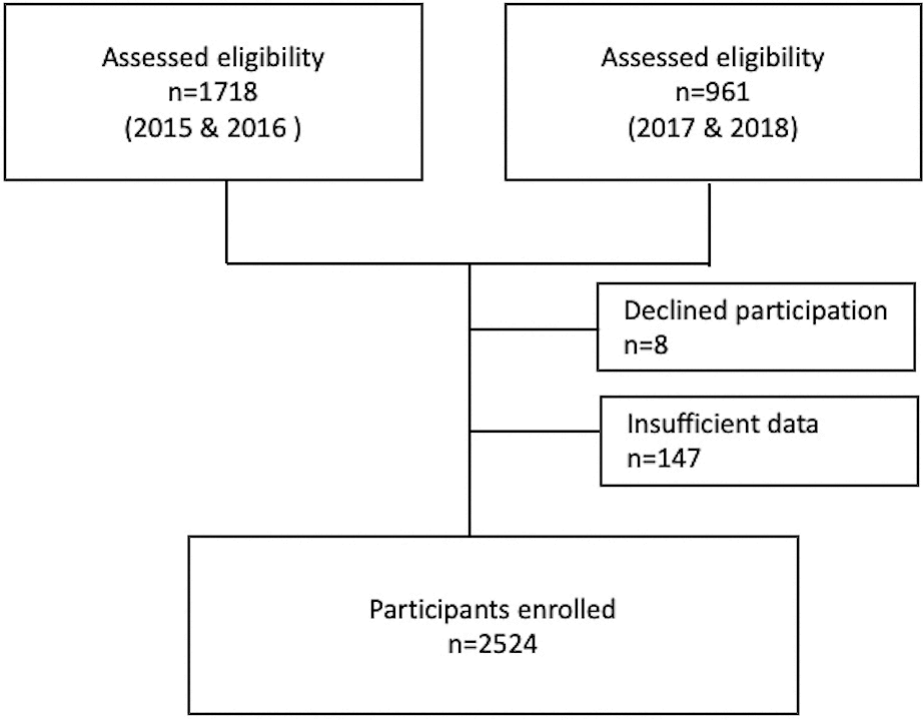
Flowchart of this study.

This experimental study aimed to clarify the effectiveness of the JPS reform in promoting scholarly activities among Japanese pediatric residents. All pediatric residents in Japan who took the pediatric board examinations during the four years from 2015 to 2018 were subjects in this study. Of all 2679 eligible participants, eight declined participation and 147 were excluded due to data omission. Consequently, 2524 trainees were enrolled (Figure 1).

### Data collection

The database information included the trainee’s sex, training duration, type of training institution, location of the institution, number of times the board examination was taken, number of research presentations and research publications, and the types of publications. Regarding the number of examinations taken, the first attempt was indicated as 1, with pediatricians who failed in their earlier attempt(s) showing 2 or a higher number. These variables of the multiple regression analysis were selected based on evidence from the existing literature ^(10), (13)^. The training institution categories included national or public university, private university, children’s, and community hospitals. The institution categories were defined previously ^(10)^. A research presentation was defined as a presentation conducted at an academic conference held by an academic society; therefore, conferences within the training institutions, such as case conferences, grand rounds, and morbidity and mortality conferences, were not included. A research publication was defined as an article published in a peer-reviewed journal, including Japanese journals published by Japanese academic societies as well as PubMed-indexed international journals.

### Analysis

We employed descriptive statistics to characterize the participants by sex, number of examination attempts, training duration, type of training institution, location of the institution, and number of presentations and publications. Multiple regression analysis was conducted for the number of research publications during the residency as outcomes. Test taking after the reform (i.e., taking the exam in 2017 or 2018) was treated as one of the independent variables of the regression model. Other independent variables included the demographic information from the residents, such as sex, number of examination attempts, type and location of the institution, and number of presentations, and were chosen based on evidence from the literature and statistical evidence ^(10), (13)^, such as improvement in model fit and results from the bivariate analysis. Model fit and possible multicollinearity of predictors were checked using standard diagnostic tools (*F*-statistic and *R*^2^). Statistical analyses were conducted using SPSS version 23.0 (IBM Corporation, Armonk, NY, USA).

### Ethical aspects

This study was approved by the Ethics Committees of both The National Center for Child Health and Development in December 2014 (No. 74) and the JPS in March 2015.

## Results

Of all 2524 participants, the number of the examinees before (2015 and 2016) and after (2017 and 2018) the reform was 1580 and 944, respectively. Among all residents throughout four years, 1018 (40.3%) were female, and the mean number of test-taking experiences was 1.5 (*SD* 0.1). Regarding the training institutions, 35.3% trained at a national/public university hospital, 24.9% at a private university hospital, 9.5% at a children’s hospital, and 30.3% at a community hospital. The geographical distribution of the participants was as follows: 24.5% were located in Tokyo, 18.1% in Kinki, 15.6% in Kanto excluding Tokyo, 14.7% in Chubu, 12.1% in Kyushu/Okinawa, 7.8% in Hokkaido/Tohoku, and 7.2% in Chugoku/Shikoku.

In scholarly activities, the residents gave 1.2 (*SD* 0.9) presentations/year and published 0.06 (*SD* 0.16) articles/year during their residency before the reform (2015 and 2016). In detail, the number of publications/year in English and Japanese was 0.02 (*SD* 0.09) and 0.04 (0.13), respectively, and the original article and case report publications/year were 0.04 (*SD* 0.13) and 0.02 (*SD* 0.07), respectively. In 2016 and 2017, after the reform, the residents gave 1.3 (*SD* 1.0) presentations/year and published 0.21 (*SD* 0.18) articles/year. The yearly publication number of English, Japanese, and original articles and case reports after the reform (2017 and 2018) was 0.05 (*SD* 0.13), 0.16 (*SD* 0.17), 0.16 (*SD* 0.17), and 0.04 (*SD* 0.11), respectively (Table 1).

**Table 1.** Demographics of the Participants.

	2015 & 2016 (*n* = 1580)	2017 & 2018 (*n* = 944)	Total (*n* = 2524)
Female sex, % (*n*)	41.1 (649)	39.1 (369)	40.3 (1018)
Test attempts (times), *M* (*SD*)	1.5 (1.0)	1.4 (1.0)	1.5 (1.0)
Types of training institution, % (*n*)
Public/National university hospital	34.5 (545)	36.5 (345)	35.3 (890)
Private university hospital	24.9 (394)	24.9 (235)	24.9 (629)
Children’s hospital	9.1 (144)	10.2 (96)	9.5 (240)
Community hospital	31.5 (497)	28.4 (268)	30.3 (765)
Location of the institution, % (*n*)			
Tokyo	24.1 (381)	25.2 (238)	24.5 (619)
Hokkaido/Tohoku	8.7 (137)	6.4 (60)	7.8 (197)
Kanto	15.0 (237)	16.6 (157)	15.6 (394)
Chubu	14.3 (226)	15.3 (144)	14.7 (370)
Kinki	18.1 (286)	18.0 (170)	18.1 (456)
Chugoku/Shikoku	7.2 (114)	7.2 (68)	7.2 (182)
Kyushu/Okinawa	12.6 (199)	11.3 (107)	12.1 (306)
Number of academic presentations/year, *M* (*SD*)	1.2 (0.9)	1.3 (1.0)	1.2 (0.9)
Total Number of research publications/year, *M* (*SD*)	0.06 (0.16)	0.21 (0.18)	0.11 (0.18)
Publication in English/year, *M* (*SD*)	0.02 (0.09)	0.05 (0.13)	0.03 (0.10)
Publication in Japanese/year, *M* (*SD*)	0.04 (0.13)	0.16 (0.17)	0.08 (0.16)
Original paper/year, *M* (*SD*)	0.04 (0.13)	0.16 (0.17)	0.09 (0.16)
Case report paper/year, *M* (*SD*)	0.02 (0.07)	0.04 (0.11)	0.03 (0.09)

A multiple regression analysis model for the research publications indicated that predictors for the examinee after the reform, i.e., sex, number of examination attempts, type and location of the institution, and research presentation experiences, explained 23% of the variance in the publication patterns (*F* (13, 2510) = 56.58, *p* < 0.001), and the standardized coefficients (*β*) of the predictors were obtained. The post-reform examinees (*β* = 0.37, *p* < 0.01) and the number of research presentations (*β* = 0.28, *p* < 0.01) was found to be significantly associated with that of research publications. While no contributive variables were found in the institution types, residents in the Kyushu and Okinawa area (i.e., southern island area in Japan) published fewer articles than those in the Tokyo area (*β* = −0.05, *p* = 0.03) (Table 2).

**Table 2.** Simultaneous Multiple Regression for Publications.

Variable	Adjusted *β*	95% CI	*p* value
Constant		−0.02 to 0.04	0.65
Examinees after the reform	0.37	0.13 to 0.15	<0.01
Male sex	−0.02	−0.02 to 0.01	0.21
Test attempts (times)	0.03	−0.001 to 0.01	0.08
Number of research presentations	0.28	0.05 to 0.06	<0.01
Types of training institution		
Public/National university hospital (Reference)	-	-	-
Private university hospital	−0.02	−0.03 to 0.01	0.43
Children’s hospital	0.01	−0.02 to 0.03	0.68
Community hospital	−0.02	−0.02 to 0.01	0.40
Location of the institution			
Tokyo	-	-	-
Hokkaido/Tohoku	−0.01	−0.04 to 0.02	0.54
Kanto	0.03	−0.01 to 0.04	0.15
Chubu	−0.01	−0.03 to 0.02	0.54
Kinki	−0.03	−0.04 to 0.01	0.18
Chugoku/Shikoku	−0.02	−0.04 to 0.02	0.50
Kyushu/Okinawa	−0.05	−0.05 to −0.002	0.03

*Note*. *R*^2^ = .227 (*p* < 0.05).

## Discussion

This is the first nationwide study investigating the effect of the policy mandating that residents publish research articles as a qualification for taking the board examination to promote their scholarly activities during residency. We found that the residents who took the board examination after implementation of the reform were significantly more productive regarding research publications, meaning that the new policy effectively facilitated scholarly activities of pediatric residents. We also found that the number of research presentations is a significant predictor of publishing during residency, indicating that presentation experience is one of the critical milestones for successful publication by pediatric residents.

A previous study using the pre-reform JPS examination database also showed that while 90% of pediatric residents gave research presentations in academic conferences, only 16% of them published research papers in peer-reviewed journals during their residency training. In addition, disparities in the residents’ research publications in peer-reviewed journals among the institution types and locations were found in the pre-reform survey ^(10)^.

The current study shows that the number of publications significantly increased and the disparity in research publications among institutions improved after the reform. Therefore, it is considered that this JPS initiative has facilitated the research activities of residents, which used to end with conference presentations, to reach publication.

Several reports from the United States indicated that female physicians’ or surgeons’ clinical performance was superior to that of male doctors ^(14), (15), (16)^; however, a gender gap was not found in the publication performance of the Japanese pediatric residents. Instead, our results showed that there is still a difference in the publication performance between the locations of the residency institutions; thus, it is necessary to implement a strategy to address the area gap in publication activity of the residents.

While residency programs in any subspecialty have sought the best strategies to address the potential barriers for completing research projects during the residency, it may be challenging to determine the one solution to deal with this challenge as every program has a different context, such as diversity of background of residents, the competencies of faculties, and available resources. The JPS tackled this issue with the mandating policy requiring research publication prior to taking the board examination, a more powerful solution. Although this prerequisite policy may be a robust way to promote pediatric scholarly activities in Japan, we need to be aware of the potential risk of the negative impacts of this reform. It is notable that the number of examinees remarkably decreased from 1580 to 944 after the reform. There may be two reasons for this change as follows: (1) many residents took the board examinations in 2015 and 2016 before the research publication became mandatory, and (2) there were many residents who did not take the examinations because they could not publish a research paper before applying for the examinations in 2017 and 2018. After this initiative has been established in several years, comprehensive evaluation for this mandatory policy would need to be assessed.

### Limitations

There are several limitations to this research. First, this study examined the policy outcomes only two years before and after its implementation; therefore, the long-term outcome is still unclear. A follow-up survey is needed to investigate the long-term impacts of this policy. Second, there is a potential for sampling and selection bias of the participants. The participants in 2017 and 2018 were the examinees of the board examinations who met the eligibility criteria for taking the test; therefore, the post-participants do not include the residents who could not fulfill the exam criteria because they did not publish a research article. While our statistical analysis by multiple regression addressed these biases, it is still necessary to investigate those residents who failed to publish a research article before taking the exam; it is possible to analyze the JPS database of examinations in the future. Third, although we revealed a disparity in publication patterns related to the location of the training hospital, the nature of the barriers to research in areas with limited scholarly activities and how the faculties there can overcome this issue are unclear. Therefore, another study including qualitative data on the residents and faculty must explore solutions to these institutional disparities. Finally, we could not assess the impact of the COVID-19 pandemic on the pediatric residents’ scholarly activity as this study was conducted before the pandemic. Further pre/post-COVID-19 pandemic studies are needed to investigate this issue.

In conclusion, the policy requiring the residents to publish research articles as a prerequisite for the board examination effectively promotes research activities of pediatric residents in Japan.

## Article Information

### Conflicts of Interest

None

### Sources of Funding

This work was supported by the grants from the National Center for Child Health and Development in Japan (grant number 26-15 & 2020B-19).

### Acknowledgement

The authors wish to thank all those who participated in this survey.

### Author Contributions

ON performed the statistical analyses and drafted the manuscript; NM supported the statistical analyses; KK and TY critically reviewed the manuscript, and AI designed this study, generated the database, and supervised the overall study process. All authors read and approved the final manuscript.

The members of the Japan Pediatric Society Steering Committee for Board Examinations: Satoru Nagata, Katsumi Nishiya, Shinichiro Sekiguchi, Hirokazu Arakawa, Koichi Kusuhara, Katsuhiko Yoshii, Toshiyuki Kitoh, Naoko Ishitoya, Hiroshi Azuma, Junichi Oki, Mika Ishige, Yasuyuki Suzuki, Akiyoshi Nariai, Yasuhiro Takeshima, Kohmei Ida, Kenji Ihara, Masao Kobayashi, Ryuta Nishikomori, Ichiro Morioka, and Masahiko Kishiro, Muneaki Matsuo.

### Approval by Institutional Review Board (IRB)

This study was approved by the Ethics Committees of both the National Center for Child Health and Development in December 2014 (No. 74) and the Japan Pediatric Society in March 2015.

## References

[1] Mink RB, Myers AL, Turner DA, et al. Competencies, milestones, and a level of supervision scale for entrustable professional activities for scholarship. Acad Med. 2018;93(11):1668-72. 29995669 10.1097/ACM.0000000000002353

[2] Nomura O, Kobayashi T, Nagata C, et al. Needs assessment for supports to promote pediatric clinical research using an online survey of the Japanese children’s hospitals association. JMA J. 2020;3(2):131-7. 33150245 10.31662/jmaj.2019-0037 PMC7590392

[3] ACGME. Common program requirements [Internet]. [cited 2021 Jul 16]. Available from: http://www.acgme.org/What-We-Do/Accreditation/Common-Program-Requirements.

[4] McHenry MS, Abramson EL, McKenna MP, et al. Research in pediatric residency: national experience of pediatric chief residents. Acad Pediatr. 2017;17(2):144-8. 28259335 10.1016/j.acap.2016.09.010

[5] Abramson EL, DiPace JI, Loughlin GM. Scholarly activity training during residency: ensuring a meaningful experience for all graduates. J Pediatr. 2018;201:5-7. e3. 30122366 10.1016/j.jpeds.2018.07.029

[6] Smiljkovic M, Chevallier M, Freycon C, et al. Lack of dedicated research time was the main barrier to French paediatric residents publishing academic papers. Acta Paediatr. 2021;110(6):1963-4. 33484010 10.1111/apa.15770

[7] Romanos-Sirakis E, Varghese S, Demissie S, et al. Pediatric research and scholarship committee: single institution initiative to enhance scholarly activity of pediatric residents. Acad Pediatr. 2020;20(7):905-9. 32302756 10.1016/j.acap.2020.04.005

[8] Tumin D, Crotty J, Aikman I, et al. Out of time? Resident scholarly publication and time pressures. Acta Paediatr. 2021;110(6):1965. 33713034 10.1111/apa.15831

[9] Chevallier M, Smiljkovic M, Cesar T, et al. Publishing during residency: not just a question of time. Acta Paediatr. 2021;110(6):1966. 33660334 10.1111/apa.15830

[10] Ishiguro A, Nomura O, Michihata N, et al. Research during pediatric residency training: a nationwide study in Japan. JMA J. 2019;2(1):28-34. 33681510 10.31662/jmaj.2018-0007 PMC7930706

[11] Nomura O, Mishina H, Kobayashi Y, et al. Limitation of duty hour regulations for pediatric resident wellness: a mixed methods study in Japan. Medicine. 2016;95(37):e4867. 27631253 10.1097/MD.0000000000004867 PMC5402596

[12] Nomura O, Mishina H, Jasti H, et al. Pediatric resident perceptions of shift work in ward rotations. Pediatr Int. 2017;59(10):1119-22. 29081080 10.1111/ped.13370

[13] Nomura O, Onishi H, Park YS, et al. Predictors of performance on the pediatric board certification examination. BMC Med Educ. 2021;21(1):122. 33618691 10.1186/s12909-021-02515-z PMC7898761

[14] Tsugawa Y, Jena AB, Figueroa JF, et al. Comparison of hospital mortality and readmission rates for medicare patients treated by male vs female physicians. JAMA Intern Med. 2017;177(2):206-13. 27992617 10.1001/jamainternmed.2016.7875 PMC5558155

[15] Tsugawa Y, Jena AB, Orav EJ, et al. Age and sex of surgeons and mortality of older surgical patients: observational study. BMJ. 2018;361:k1343. 29695473 10.1136/bmj.k1343 PMC5915700

[16] Tsugawa Y, Jha AK, Newhouse JP, et al. Variation in physician spending and association with patient outcomes. JAMA Intern Med. 2017;177(5):675-82. 28288254 10.1001/jamainternmed.2017.0059 PMC5470365

